# Impact of soil compaction degree modulated by initial water content on maize root phenotype and hydraulic properties

**DOI:** 10.3389/fpls.2026.1764502

**Published:** 2026-02-11

**Authors:** Yuanyuan Fu, Zhuanyun Si, Shoutian Ma, Yahui Cheng, Zhandong Liu, Yang Gao

**Affiliations:** 1 Key Laboratory of Crop Water Use and Regulation, Ministry of Agriculture and Rural Affairs, Institute of Farmland Irrigation, Chinese Academy of Agricultural Sciences, Xinxiang, China; 2 Graduate School of Chinese Academy of Agricultural Sciences, Beijing, China; 3 School of Hydraulic and Civil Engineering, Ludong University, Yantai, Shandong, China

**Keywords:** compaction, maize, root hydraulic characteristics, root phenotype, soil water content

## Abstract

**Introduction:**

Mechanical compaction limits crop production potential by disrupting soil structure and inhibiting root growth. To achieve precise and sustainable agricultural management, it is necessary to clarify the response patterns of compaction effects on root development and water conduction under different initial moisture contents.

**Methods:**

In this study, *Zheng58* and *Chang7-2*, were subjected to a compaction force of 350 kPa under two initial moisture levels of 40% and 80% of field capacity (FC). The effects of compaction on root morphology, aerenchyma formation, and root hydraulic conductivity were investigated.

**Results and discussion:**

The results show that soil compaction significantly affected root morphology, aerenchyma formation, and hydraulic conductivity of maize seedlings, and these effects depended on the initial soil moisture at the time of compaction. Regarding root morphology, compaction increased root diameter in both, maize cultivars by 11.6%-43.2%, but at 80% FC, root length was significantly reduced by approximately 72%, and the proportion of fine roots decreased by 5.0%-6.3%. For aerenchyma area, compaction substantially promoted the expansion of aerenchyma area the root-shoot junction, with the most pronounced increase observed at 40% FC. The effect of soil compaction on theoretical root hydraulic conductivity at both the 2.5cm root apex and the root-shoot junction was strongly influenced by the soil moisture at the time of compaction: under 40% FC, theoretical root hydraulic conductivity significantly increased, whereas at 80% FC, it decreased by 67.1%-78.9% at the apex and 40.2%-41.4% at the root-shoot junction. These results indicate that high soil moisture exacerbates the inhibitory effect of compaction on root hydraulic function, highlighting the importance of managing soil moisture to mitigate compaction stress.

## 1 Introduction

Soil compaction refers to the process that under the action of external pressure, particles in unsaturated soil rearrange closely, soil porosity decreases and soil bulk density increases (Soil Science Society of America, 1997). During the process of modern agricultural development, the continuous improvement of mechanization levels has brought numerous conveniences and efficiencies to agricultural production. However, this is accompanied by the large-scale and heavy-duty development of agricultural machinery equipment and a significant increase in operation frequency, which causes large and heavy agricultural machinery to exert continuous high-intensity pressure on the soil when operating frequently in the fields. Consequently, soil compaction has become increasingly prominent and now represents a critical issue that cannot be overlooked in contemporary agricultural practices (Keller et al., 2019). Globally, the area of land degraded by soil compaction amounts to approximately 68 million hectares (Oldeman et al., 1991). However, this figure has likely increased considerably at present. In China, field investigations have revealed that the Guanzhong Plain (Zhu et al., 2014) and North China Plain (Wu et al., 2022) have experienced varying degrees of soil compaction. The Northeast Black Soil Region (Lin et al., 2022; Zhang et al., 2006), due to its originally lower soil bulk density, faces a relatively higher potential risk of soil compaction under external pressure. Meanwhile, the southern red soil region also suffers from soil compaction (Li et al., 2024). After soil compaction, the air content between soil particles diminishes, soil porosity decreases, and bulk density increases (Augustin et al., 2020; Frene et al., 2024). This alteration triggers a cascade of adverse consequences: on one hand, the water permeability and soil aeration deteriorate, exacerbating surface runoff formation; on the other hand, critical nutrients such as carbon and nitrogen become more prone to leaching, while crop nutrient uptake efficiency declines. Collectively, these effects ultimately impair crop productivity (Hargreaves et al., 2019; Obour and Ugarte, 2021). Canarache et al. (1984) demonstrated that in most of the experimental regions, maize grain yield exhibited a linear decline of 13 kg·ha^-1^ for every 1 kg·m^-3^ increase in soil bulk density. Thus, mechanical soil compaction has become a critical factor contributing to soil degradation, environmental deterioration of soil systems, and crop yield reduction (Castioni et al., 2021; Shah et al., 2017). However, the impact of soil compaction on crop growth exhibits dual effects: Moderate compaction can promote plant growth by reducing non-capillary pores to stabilize soil moisture, improving emergence rates, enhancing root water and nutrient uptake efficiency (Bello-Bello et al., 2022; Frene et al., 2024); whereas excessive compaction significantly increases soil bulk density, reduces aeration, inhibits root development and nutrient absorption, ultimately leading to yield reduction (Kuht and Reintam, 2004; Liu et al., 2022; Mcnabb et al., 2001).

The degree of soil compaction is predominantly influenced by soil factors including soil texture, organic matter content, bulk density, aggregate stability, tillage practices, and soil moisture content (Alakukku et al., 2003; Hamza and Anderson, 2005). However, soil moisture content critically influences soil strength and determines its susceptibility to mechanical compaction, high soil moisture reduces the soil’s load-bearing capacity, thereby increasing compaction risks during operations on poorly drained fields (Alakukku et al., 2003; Hamza and Anderson, 2005).

Currently, researchers worldwide have conducted extensive studies on the effects of initial soil moisture content on soil physical properties. Xiong et al. (2020) found that soil compaction under 80% FC conditions resulted in significantly lower porosity compared to compaction treatments under 60% FC conditions. Medvedev and Cybulko (1995) showed that under higher soil moisture, the soil’s permissible bearing capacity significantly decreases; however, under low soil moisture, even under maximum load, soil deformation depth does not exceed 2 cm. Studies have shown that soil moisture content significantly influences the soil’s compressive strength, which governs its susceptibility to mechanical compaction (Alakukku et al., 2003; Hamza and Anderson, 2005; Long et al., 2024). Moreover, the same study reported that elevated moisture levels reduce shear strength and load-bearing capacity, thereby increasing compaction risks.

Mechanical compaction increases mechanical impedance during crop growth, restricting root elongation and resulting in shallower root systems (Keller et al., 2019). Concurrently, studies have demonstrated significant interspecific and varietal differences in tolerance to soil compaction among crops, with such variation strongly linked to their root architectural traits and acclimation strategies (Correa et al., 2022; Xiong et al., 2020; Yue et al., 2021). Root systems, as the organs through which crops absorb water and nutrients from the soil, directly influence crop growth, development, and yield formation through their morphological structure and physiological functions (Fu et al., 2024). Based on this, elucidating the effects of soil compaction on root morphological and hydraulic traits of maize varieties under varying initial soil moisture conditions not only advances theoretical understanding of crop root adaptation mechanisms but also provides a scientific basis for precision field management. In this study, compaction treatments were applied under different initial moisture contents to systematically analyze the responses of maize root morphological parameters, anatomical structures, and hydraulic traits to compaction. The findings establish a robust theoretical foundation for precision field management.

## 2 Materials and methods

### 2.1 Experimental soil

The experimental black soil was collected from Hongxing Farm (48°19′N, 127°08′E) in Beian City, Heilongjiang Province, with sampling depth ranging from 0–20 cm. After natural air-drying, plant roots and impurities were removed, and the soil was crushed and sieved through a 4-mm mesh. The soil had a pH of 5.6, electrical conductivity (EC) of 219.2 μS·cm^-^¹, organic carbon content of 76.9 g·kg^-^¹, alkali-hydrolyzable nitrogen of 324.2 mg·kg^-^¹, available potassium of 377.1 mg·kg^-^¹, available phosphorus of 79.4 mg·kg^-^¹, total nitrogen of 2.5 mg·g^-^¹, and total phosphorus of 5.1 mg·g^-^¹. The experiment was conducted in 2024 in a controlled-climate chamber at the Qiliying Comprehensive Experimental Base (35°09′N, 113°48′E) of the Chinese Academy of Agricultural Sciences (CAAS) in Henan Province. During the trial, the photosynthetic photon flux density (PPFD) was 600μmol/(m²·s) with a 12 h/12 h day/night photoperiod, temperature regimes of 28 °C (day)/20 °C (night), and relative humidity maintained at 40-50%.

### 2.2 Experimental design

**Figure 1:**
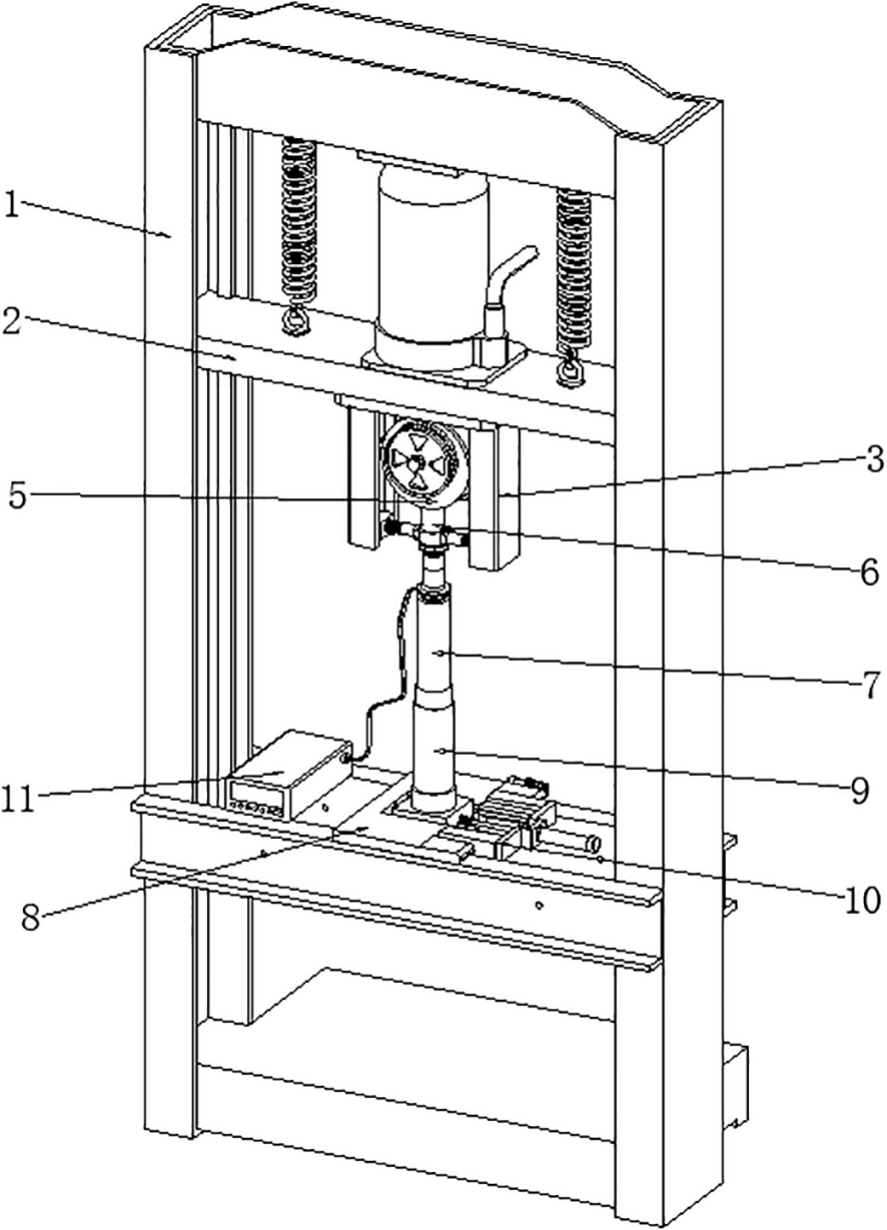
Compacting device (1. installation of vertical frame; 2. sliding frame plate; 3. installation frame; 4. installation column; 5. rotating sleeve frame; 6. connecting column; 7. extrusion column; 8. support frame plate; 9. container; 10. rotating frame; 11. Detector ontology).

Two maize cultivars, Zheng 58 and Chang 7-2, were used in this experiment. For each cultivar, and uncompacted control treatment was established (CK1 for Zheng 58 and CK2 for Chang 7-2). Soil compaction treatments were applied at two initial soil moisture levels prior to compaction: 40% and 80% of field capacity (FC). Specifically, T1 and T2 represent compaction treatments for Zheng 58 at initial soil moisture levels of 40% FC and 80% FC, respectively, while T3 and T4 represent the corresponding compaction treatment for Chang 7-2. Soil physical properties were measured immediately after compaction and before sowing. At this stage, no crop had been planted; therefore, CK1 and CK2 treated as a single uncompacted control (CK) for soil physical analyses. After compaction, soil moisture in all treatments, including controls, was adjusted and maintained at 75% FC for sowing and throughout the entire experimental. After adjusting to the target moisture content, the sieved soil was homogenized and packed into PVC cylinders (height: 15 cm, internal diameter: 7 cm). The soil compaction stress induced per field pass was calculated as 350 kPa based on the specifications of local agricultural machinery models. The soil was compacted using a self-made compaction device (**Figure 1**). Prior to the application of soil compaction, soil bulk density inside all PVC cylinders was standardized at 1.3 g cm^3^ to ensure uniform initial soil packing conditions.

After compaction, the initial soil moisture content was adjusted to 75% FC via manual watering. Soil moisture in all treatments was maintained at 75% *FC* pre-sowing and throughout the experiment. Uniform-sized, plump seeds were surface-sterilized by soaking in 1% (v/v) sodium hypochlorite for 20 min, followed by rinsing with distilled water several times. Disinfected seeds were sown in PVC cylinders, with one seed per cylinder. For each treatment, 10 independent soil columns (PVC cylinders) were established as biological replicates. Due to the destructive nature of measurements, different subsets of cylinders were used for specific analyses, with n=3 biological replicates for root morphological traits, root-soil water potential difference and bleeding sap measurements, and root anatomical observations, respectively. To minimize soil evaporation, a 2-cm-thick layer of quartz sand (2-mm diameter) was spread on the soil surface post-germination. At 10 days after sowing (DAS), when roots had grown to the cylinder base, samples were collected for subsequent analyses.

### 2.3 Measurement of indicators

#### 2.3.1 Measurement of soil bulk density, porosity and penetration resistance

Weigh the empty ring knife as *W1*. Following the experimental design, after packing the soil into the ring knife, place the sample in an oven at 105 °C to dry until constant weight. Allow the sample to cool naturally to ambient temperature, then measure the combined mass of the ring knife and dried soil, recorded as *W2*. Soil penetration resistance was measured using an Eijkelkamp 6.0 penetrometer. The formulas for bulk density and porosity are as follows (Equations 1, 2):

(1) $$\gamma =\frac{\left(W2-W1\right)}{V}$$

(2) $$P=(1-\frac{\gamma }{d})\times 100$$

where *γ* represents soil bulk density (g·cm^-^³); *W_1_* is the mass of the ring knife (g); *W_2_* is the total mass of the ring knife and soil (g); *V* is the volume of the ring knife (100 cm³); *P* denotes soil porosity (%); and *d* is soil particle density, set to 2.65 g·cm^-^³.

#### 2.3.2 Measurement of root-soil water potential difference and root bleeding fluid

On the 10th day after sowing, three pots of corn with uniform growth were selected from each treatment. The primary stems were surgically severed 1 cm above ground level at 19:00 (before lights were turned off) using sterilized dissection tools, immediately secured with absorbent cotton pads over the excision site, and hermetically enclosed with pre-weighed parafilm sealing film (initial combined mass recorded as *W_1_)*. The post-treatment weight (*W_2_*) of absorbent cotton and sealing film was gravimetrically recorded at 07:00 the following morning, with the derived differential (*W_2_*−*W_1_*) quantitatively representing the bleeding sap exudation from excised maize plants during the 12-hour monitoring phase. Root systems and bulk soil from respective treatments were systematically collected. Prior to analysis, the WP4C instrument (Decagon Devices, Pullman, WA, USA) was preheated for 30 minutes in a temperature-controlled (25 °C), draft-free environment and calibrated using standard calibration solutions. During measurement, root samples were cut into fragments and evenly positioned in dedicated chambers for root water potential determination, while soil samples were analyzed using soil-specific cassettes for soil water potential assessment. The root-soil water potential difference was calculated from root and soil water potentials.

#### 2.3.3 Measurement of root system parameter

Following completion of root bleeding sap quantification, excised root systems were thoroughly rinsed to remove adhered particulates. Subsequent morphometric characterization was performed using an EPSON Perfection V800 scanner (Epson China Co., Ltd., Shanghai) under standardized illumination (600 dpi resolution, 24-bit color depth). Quantitative architectural parameters-including total root length, mean root diameter, root surface area, root volume, number of root tips, branching number-were algorithmically quantified via WinRHIZO Pro v7.4.2 (Regent Instruments Inc., Québec, Canada) using pre-validated segmentation thresholds (Fu et al., 2024).

#### 2.3.4 Measurement of root anatomical architecture and hydraulic conductivity

Each treatment included three maize plants randomly selected, and their root systems were thoroughly cleaned using deionized water. For each plant, one primary root with a comparable diameter and without visible mechanical damage was selected to ensure anatomical consistency. Representative root segments were uniformly excised at two fixed positions on each primary: 2.5 cm distal from root apex and root-shoot transition zones, using sterile surgical blades. These positions were selected to minimize anatomical heterogeneity along the root axis and ensure comparability among treatments. For each position, the root segment from each plant was sectioned into multiple transverse sections, and one anatomically intact cross section was randomly selected for quantitative analysis. Thus, each position included three biological replicates per treatment. Excised tissues were immediately immersed in formaldehyde-acetic acid-ethanol (FAA) fixative solution (v/v = 5:5:90) for 48-hour immersion at 4 °C. Following fixation, specimens underwent standardized paraffin embedding protocol (Leica EG1150H embedding station) and sectioned at 10 µm thickness using a rotary microtome (Leica RM2235, Wetzlar, Germany). Safranin-fast green dual staining was systematically applied to differentiate lignified vasculature from parenchymatous tissues. Permanent slides were mounted using neutral balsam (Sigma-Aldrich) under controlled humidity (40% RH). Brightfield micrograph acquisition was conducted using a Nikon Eclipse E100 upright research microscope (Nikon Instruments, Tokyo) equipped with a DS-Fi3 CMOS camera system, employing a 40× Plan Fluor objective lens (NA 0.75). Digital image calibration was performed using a stage micrometer (NIS-Elements D 5.11, Nikon). Root diameter (µm), vessel diameter (µm), cortical thickness (µm), and aerenchyma area (cm²) were measured using ImageJ32(NIH, USA). Based on the anatomical parameters obtained from transverse root sections, theoretical root hydraulic conductivity was estimated using the Hagen-Poiseuille model (Tyree and Ewers, 1991), assuming laminar flow through cylindrical xylem conduits. Specific formulas see Equation 3:

(3) $$Kxylem=\frac{m}{128\eta }\Sigma_{i=1}^{n}D_{i}^{4}$$

where *n* represents the dynamic viscosity of water (0.90 × 10^-6^ kPa); *D* denotes the vessel diameter (µm); *n* indicates the number of conducting vessels.

Xylem vessel cross-sections were geometrically approximated as ellipses in the revised Hagen-Poiseuille formulation, accounting for anatomical deviations from perfect cylindrical symmetry, with the modified equation expressed as (Correa et al., 2022). Specific formulas see Equation 4:

(4) $$D_{i}^{4}=\frac{d_{max}^{3}d_{min}^{3}}{8d_{max}^{2}+8d_{min}^{2}}$$

where *d^max^* and *d^min^* denote the maximum and minimum diameters of the xylem vessel lumen, respectively.

### 2.4 Data analysis

Preliminary data processing was executed in Microsoft Excel 2019 (Microsoft Corporation, Redmond, WA, USA). Statistical analyses were performed using DPS v13.5 (Hangzhou Reijing Information Technology Co., Ltd., Hangzhou, China; http://www.dpsw.cn). Treatment-specific differences were evaluated through Duncan’s multiple range test at a significance threshold of *P* < 0.05. Data visualization was implemented in Origin 2022 Student Version.

## 3 Result

### 3.1 Soil physical properties

**Figure 2:**
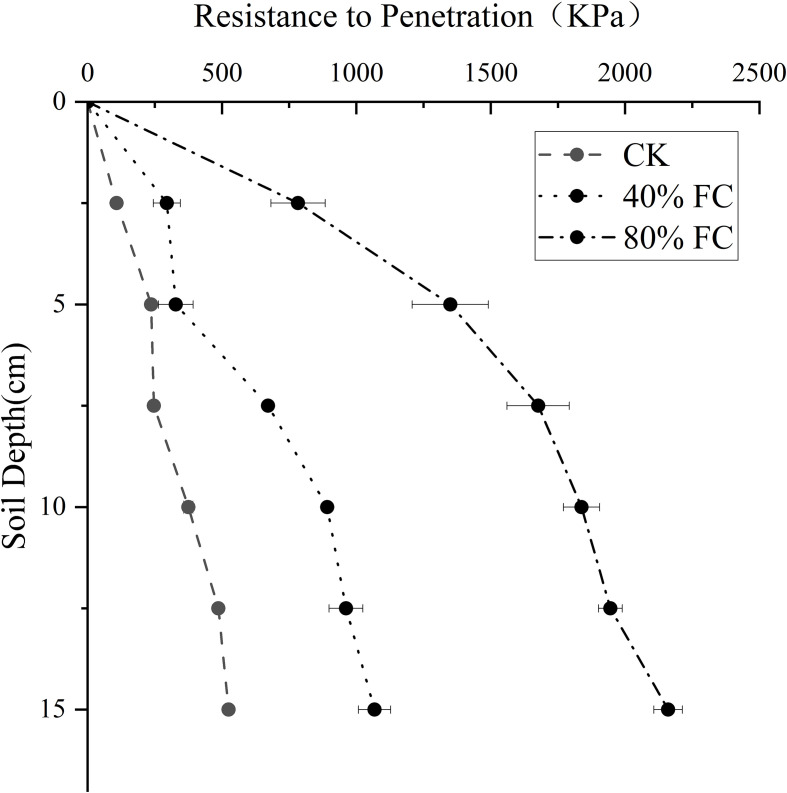
Soil penetration resistance after compaction. Values are means ± *SE* (n = 3 biological replicates).

As delineated in **Table 1**, initial moisture content significantly modulated soil compaction characteristics. Following compaction at initial soil moisture levels of 40% and 80% field capacity (FC), soil bulk density increased by 6.6% and 23.1% (*P* < 0.01), respectively, compared with the uncompacted control (CK). Correspondingly, total porosity decreased by 5.6% and 19.4%, while FC declined by 3.0% and 14.3% relative to uncompacted soil. Soil penetration resistance increased progressively with depth across all treatments (**Figure 2**). At the 5 cm soil depth, soil compacted at 80% FC exhibited a penetration resistance of 1350 kPa, which was significantly higher than that of both the uncompacted control and soil compacted at 40% FC (*P* < 0.05). In contrast, penetration resistance under the 40% FC treatment did not differ significantly from that of the uncompacted control (328 kPa vs. 237 kPa, *P*>0.05). These results indicate that soil compaction effects are strongly dependent on initial soil moisture and are substantially alleviated under lower moisture conditions.

**Table 1:** Physical properties of soils under different treatments.

Treatment	Bulk density	Total porosity	Field capacity
	g cm^3^	%	%
CK	1.2 c	54.3 a	26.8 a
40% FC	1.3 b	51.3 b	26.0 b
80% FC	1.5 a	43.8 c	23.0 c

Different lowercase letters indicate significant differences at the 0.05 level among different treatments.

### 3.2 Root morphological characteristics

**Figure 3:**
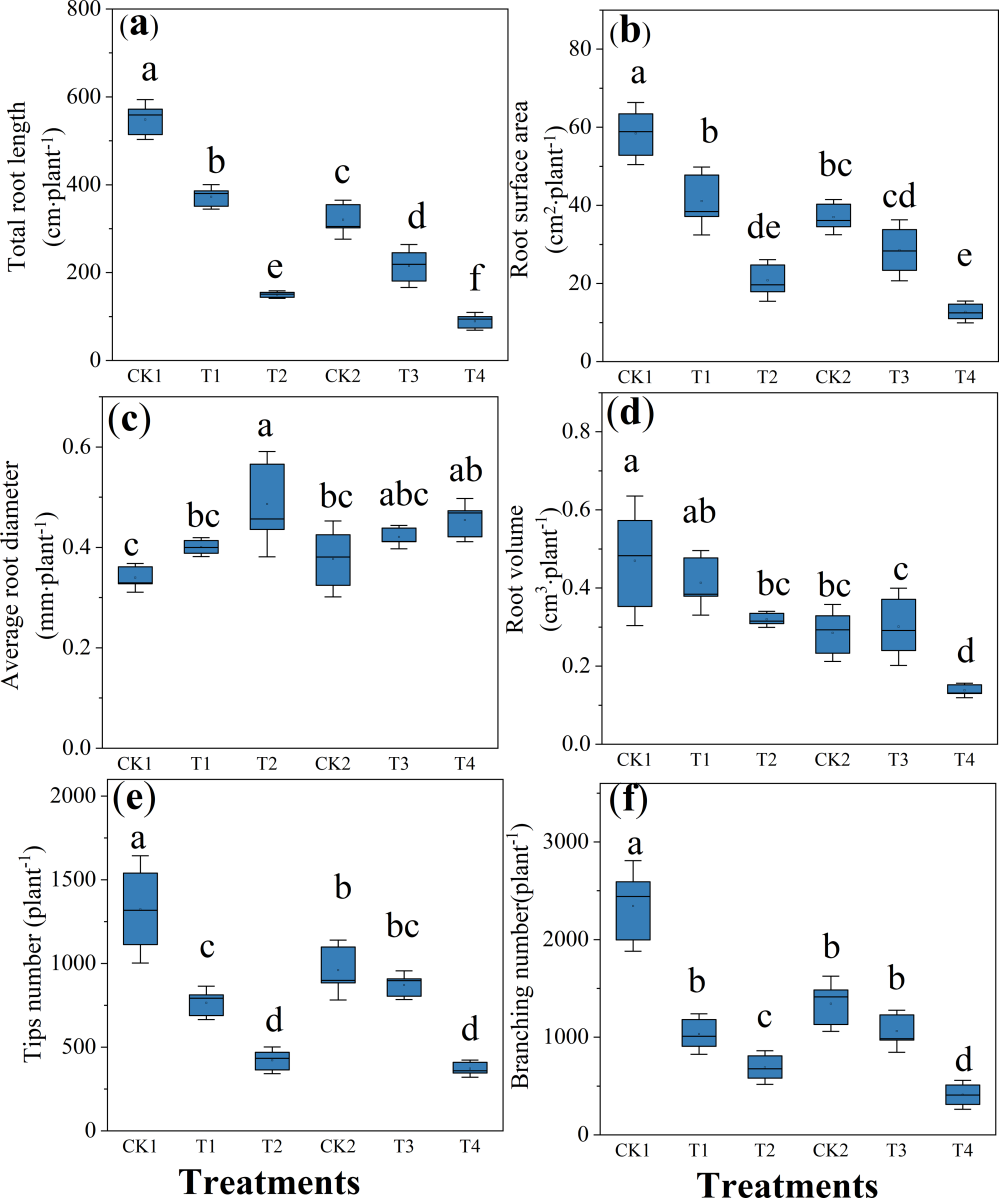
Effects of compaction on root parameters under different initial soil water content. **(a)** Total root length. **(b)** Root surface area. **(c)** Average root diameter. **(d)** Root volume. **(e)** Tips number. **(f)** Branching number. For Zhengdan 58, T1 and T2 were compared with the uncompacted control CK1; for Chang 7-2, T3 and T4 were compared with the corresponding control CK2. All comparisons were conducted within each cultivar. Data are means ± SE (n = 3). Different letters indicate significant differences among treatments within the same cultivar (P < 0.05).

As illustrated in **Figure 3**, both maize cultivars (Zheng 58 and Chang 7-2) exhibited comparable responses patterns to soil compaction across root morphological parameters, including root length, surface area, volume, average diameter, tip number, and branching frequency. Overall, soil compaction inhibited root system development, as evidenced by substantial reductions in root length, surface area, volume, tip number, and branching frequency, accompanied by a significant increase in average root diameter. In Zheng 58 (CK1), soil compaction significantly reduced root length by 32.1% and 72.6% under T1 and T2 treatments, respectively, with corresponding decrease root surface area (29.6%-64.4%) and root volume(12.0%-31.9%). Root branching frequency declined by 55.9%-70.6%, while average root diameter increased by 18.0%-43.2% compared with CK1. In Chang 7-2 (CK2), T3 and T4 treatments similarly suppressed root growth, resulting in pronounced reductions in root length, surface area, and volume. Under the T3 treatment, however, the reduction in root tip number was slight(9.4%), and branching frequency decreased by 20.9%(*P*>0.05). Concurrently, a compensatory radial growth response was observed, with average root average diameter increasing by 11.6% and 20.5% under T3 and T4, respectively, relative to the CK2.

**Figure 4:**
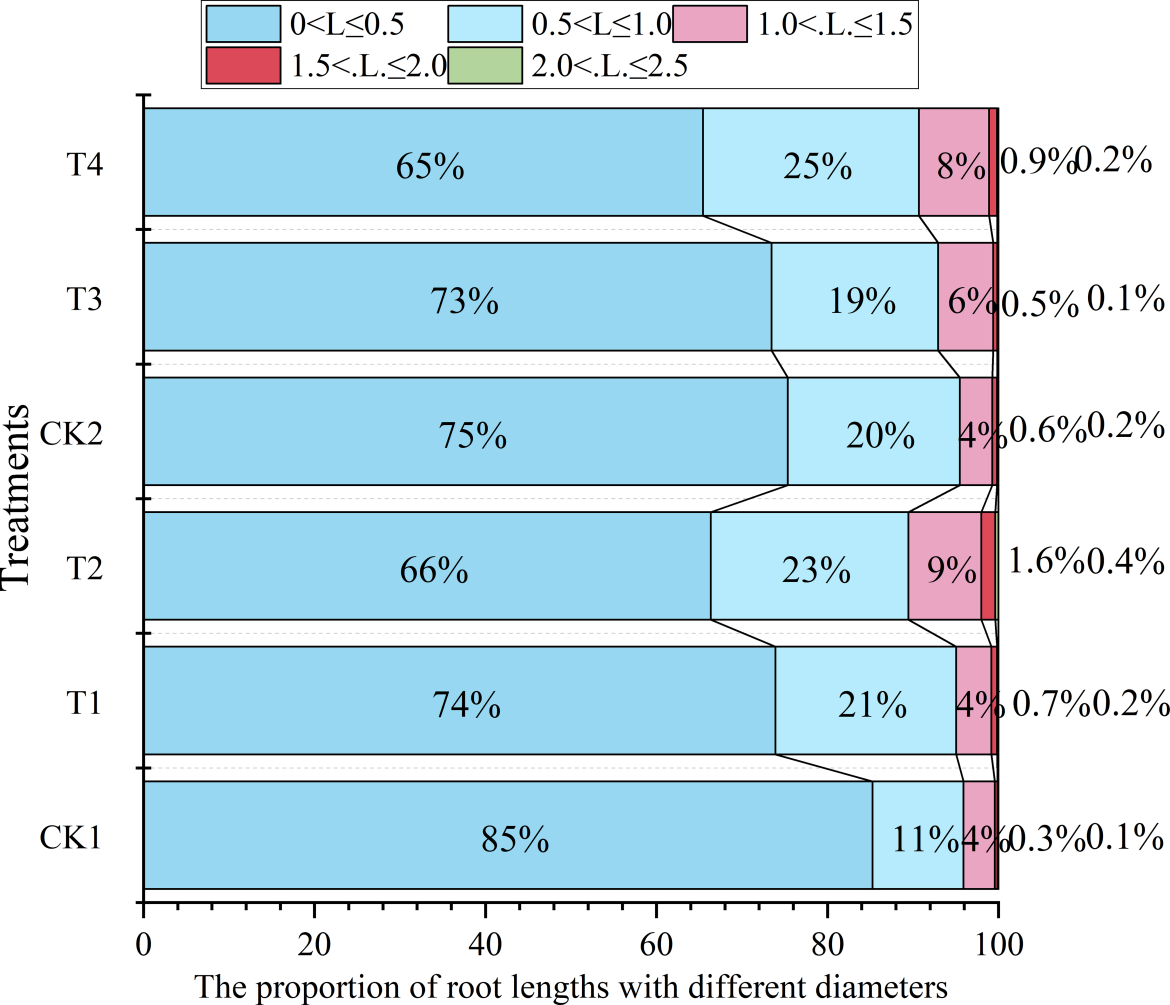
The effect of compaction on the proportion of root length with different diameter under different initial soil water content. For Zhengdan 58, T1 and T2 were compared with the uncompacted control CK1; for Chang 7-2, T3 and T4 were compared with the corresponding control CK2. All comparisons were conducted within each cultivar. Data are means ± SE (n = 3). Different letters indicate significant differences among treatments within the same cultivar (P < 0.05).

As quantified in **Figure 4**, soil compaction altered root diameter class distribution in both maize cultivars. Relative to CK1, T1 and T2 treatments reduced the proportion of fine roots (<1 mm diameter) by 1.0% and 6.3% respectively, while T3 and T4 decreased fine root fractions by 2.8% and 5.0% compared with CK2. Conversely, the proportion of coarse roots (>1 mm) increased markedly under compaction, with increases of 20.8% (T1) and 112.2% (T2) relative to CK1, and 56.3% (T3) and 105.8% (T4) relative to CK2. Across treatments, Chang 7–2 consistently maintained a higher proportion of fine roots (<1 mm diameter) and a lower allocation to coarse roots (>1 mm) compared to Zheng 58 under comparable compaction treatments.

### 3.3 Root anatomical architecture

**Figure 5:**
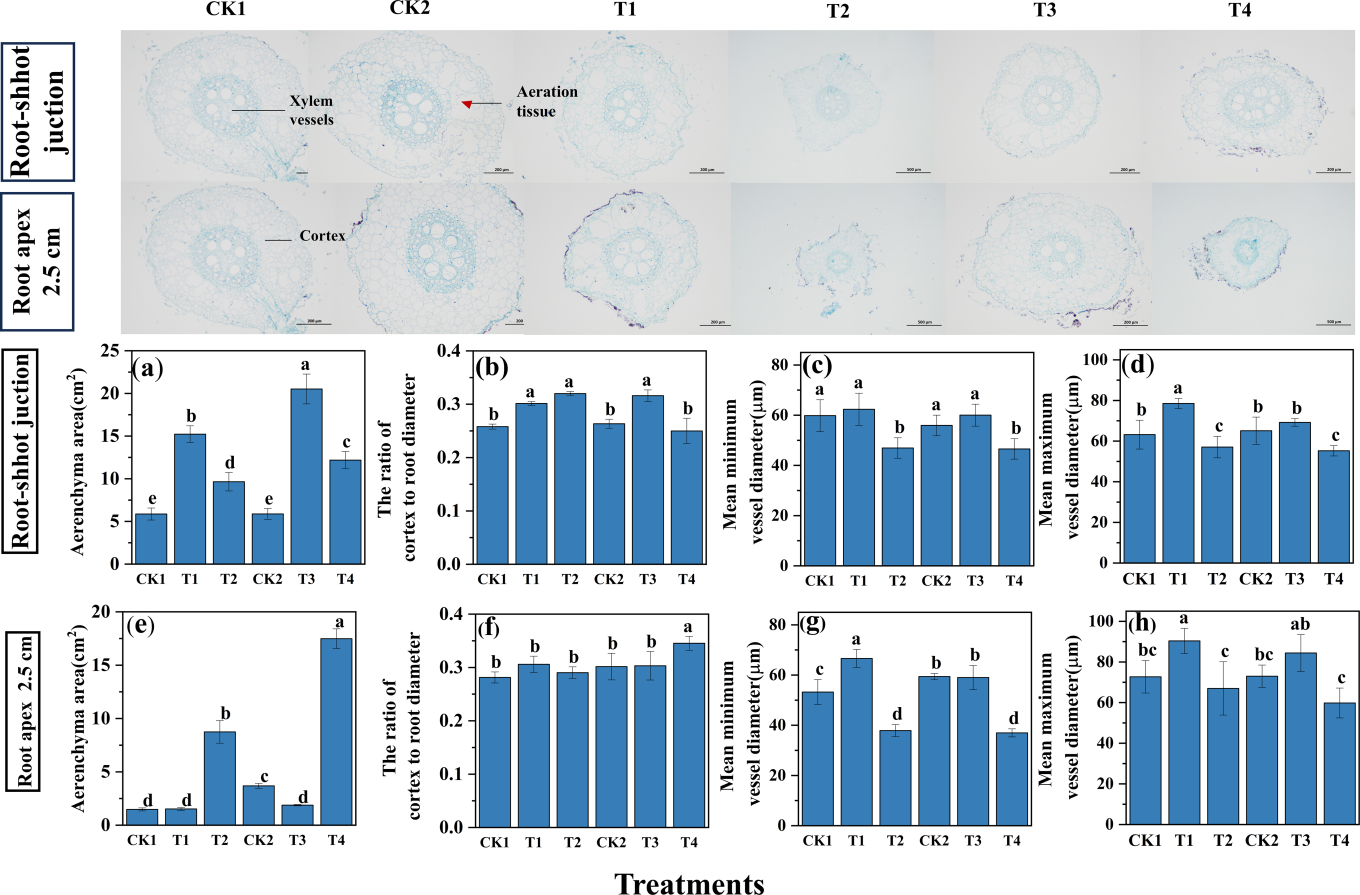
The effect of compaction on the proportion of root length with different diameter under different initial soil water content. **(a, e)** Aerenchyma area. **(b, f)** The ratio of cortes to root diameter. **(c, g)** Mean minimum vessel diameter. **(d, h)** Mean maximum vessel diameter. For Zhengdan 58, T1 and T2 were compared with the uncompacted control CK1; for Chang 7-2, T3 and T4 were compared with the corresponding control CK2. All comparisons were conducted within each cultivar. Data are means ± SE (n = 3). Different letters indicate significant differences among treatments within the same cultivar (P < 0.05).

As shown in **Figure 5**, soil compaction significantly altered aerenchyma development and vascular traits in maize roots. Relative to CK1, T1 and T2 treatments increased aerenchyma area at root-shoot junctions by159.3% and 64.4%, respectively, while changes at the root apex regions were comparatively variable. Relative to CK2, aerenchyma area at root-shoot junctions increased by 248.8% and 107.1% under T3 and T4, respectively, whereas apical aerenchyma exhibited contrasting responses between compaction intensities. As illustrated in **Figures 5B, F**, compaction at 40% initial soil moisture increased cortical proportion at the root-shoot junction by 16.8% in Zheng 58 and 20.0% in Chang 7-2, with relatively minor changes at the apical regions. Under 80% initial moisture content, cultivar-specific differences became evident, with Zheng 58 showing a greater increase in cortical proportion at the root-shoot junction, while Chang 7–2 exhibited more pronounced changes in apical cortical development. Compaction also affected vascular characteristics at the root apex. Compared with CK1, T1 increased the mean maximum and minimum vessel diameters by 24.3% and 25.2%, respectively, whereas T2 reduced both parameters. In Chang 7-2, T3 slightly altered vessel diameter, while T4 markedly reduced both maximum and minimum vessel dimensions relative to CK2.

### 3.4 Root hydraulic conductivity

**Figure 6:**
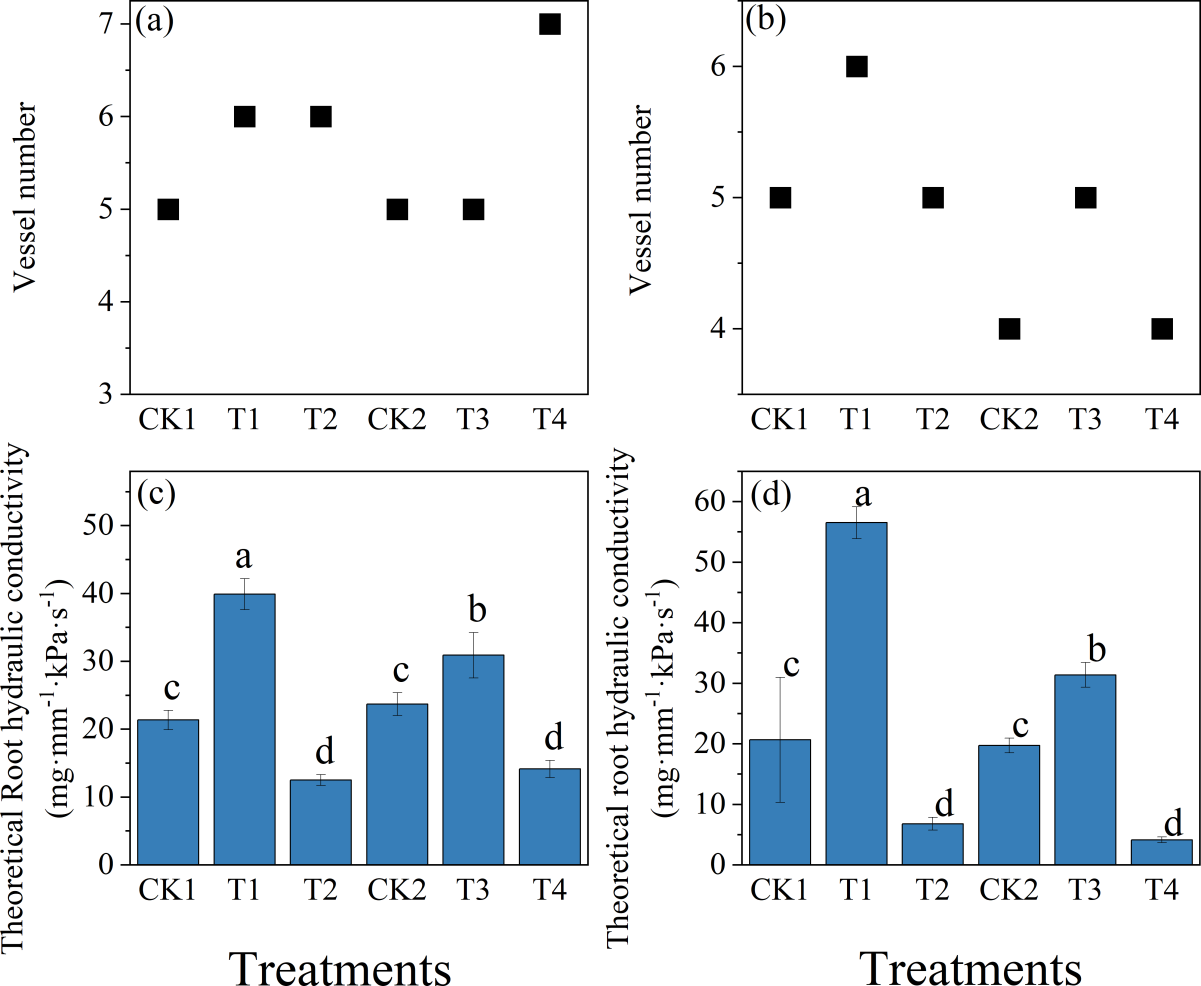
Effects of soil compaction under different initial moisture conditions on root hydraulic conductivity and xylem anatomical traits of two maize cultivars. **(a,b)** Variation in xylem vessel frequency at the root–shoot junction and 2.5 cm root apex regions. **(c,d)** Theoretical root hydraulic conductivity measured at the root–shoot junction and 2.5 cm root apex regions. For Zhengdan 58, T1 and T2 were compared with the uncompacted control CK1; for Chang 7-2, T3 and T4 were compared with the corresponding control CK2. All comparisons were conducted within each cultivar. Data are means ± SE (n = 3). Different letters indicate significant differences among treatments within the same cultivar (P < 0.05).

As shown in **Figure 6**, treatment effects on theoretical root hydraulic conductivity were evaluated relative to the cultivar-specific controls. In Zheng 58, T1 significantly increased theoretical root hydraulic conductivity at the root-shoot junction and the 2.5 cm root apex by 86.8%(*P* < 0.05) and 173.6%(*P* < 0.05), respectively, compared with CK1, whereas T2 resulted in reductions of 41.4% and 67.1%(*P* < 0.05). In Chang 7-2, T3 enhanced theoretical root hydraulic conductivity at the corresponding positions by 30.3% and 59.0%(*P* < 0.05) relative to CK2, while T4 significantly decreased conductivity by 40.4% and 78.9%.

Theoretical root hydraulic conductivity was strongly associated with internal anatomical traits, with larger xylem vessel diameter, higher vessel density, and more compact spatial arrangement collectively contributing to enhanced water transport capacity. Regarding the effects of compaction on apical vessel frequency, all treatments exhibited highly consistent variation patterns (**Figure 6B**). Notably, T4 treatment roots showed an increased vessel number accompanied by reduced mean vessel diameter (**Figure 6A**).

### 3.5 Root-soil water potential difference and root bleeding

**Figure 7:**
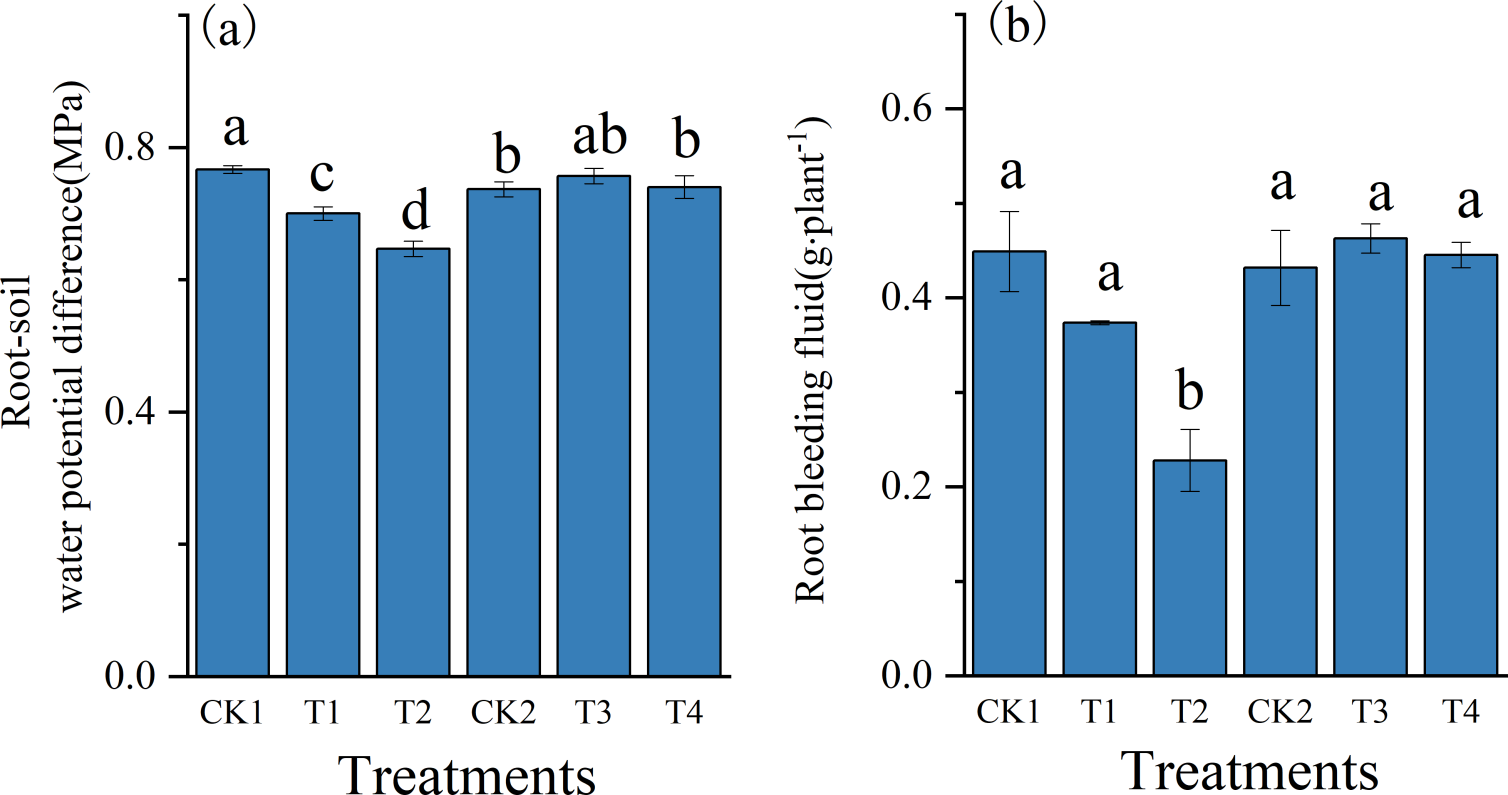
Effect of compaction on root-soil water potential difference and root bleeding under different initial soil water content. **(a)** Root-soil water potential difference. **(b)** Root bleeding fluid. Values are means ± SE (n = 3 biological replicates).

As demonstrated in **Figure 7**, soil compaction significantly altered root-soil water potential gradients and xylem exudation across treatments. Relative to CK1, T1 and T2 treatments reduced the root-soil water potential gradient by 8.7% and 15.7%, respectively (*P* < 0.05, **Figure 7A**). In contrast, T3 and T4 treatments resulted in slight increases in the gradient by 2.7% and 0.5%, respectively, compared with CK2. Compaction also reduced xylem bleeding sap volume in Zheng 58, with decrease of 16.8% and 49.2% under T1 and T2, respectively, relative to CK1 (*P* < 0.05 **Figure 7B**). Across treatments, Chang 7–2 consistently exhibited higher root-soil water potential gradients and greater xylem exudation than Zheng 58.

## 4 Discussion

The proliferation of agricultural mechanization and intensification of multi-pass field operations have significantly exacerbated soil structural degradation. This phenomenon manifests as reduced soil porosity, elevated tillage resistance, and restricted root architectural development, collectively compromising crop yield and quality (Frene et al., 2024). This study demonstrated that soil compaction intensity escalates with increasing soil moisture content, resulting in elevated soil bulk density and concomitant reduction in total porosity (**Table 1**). Further investigation revealed that compaction significantly reduced maize root length, surface area, volume, tip count, and branching frequency, while increasing mean root diameter (**Figure 3**). This morphological shift originated from compaction-induced soil structural alterations: increased soil density, reduced porosity, and elevated mechanical resistance collectively restricted root growth space, leading to shortened roots and diminished root volume. Radial expansion of cortical cells in elongation zones contributed to diameter enlargement, whereas suppressed lateral root initiation reduced tip count and branching frequency. Fewer root tips diminished soil exploration capacity, thereby impairing resource acquisition efficiency (Atwell, 1988; Clark et al., 2008). The results were consistency with previous studies by Grzesiak et al. (2013) and Potocka and Szymanowska-Pulka (2018). Furthermore, this study revealed that soil compaction significantly reduced the proportion of fine roots (<1 mm diameter) while increasing coarse roots (>1 mm) across two maize cultivars (**Figure 4**). The diminished fine root allocation impaired root water acquisition capacity, as fine roots critically enhance rhizosphere exploration through their high surface area-to-volume ratio, synergistic osmotic-capillary interactions, and adaptive plasticity under edaphic stress, thereby optimizing hydraulic conductivity (Sun et al., 2024).

The experimental results demonstrated that moderate compaction increased root aerenchyma formation, whereas intensified compaction reduced aerenchyma area (**Figures 5A, E**). Enhanced aerenchyma development correlated with reduced root metabolic expenditure, thereby improving nutrient allocation to shoots (Lynch et al., 2024). Under compaction stress, roots exhibited dual adaptive strategies: (1) Enhanced cellular activity and meristematic differentiation were observed, with apical meristem expansion and elongation zone cell turgor pressure elevation collectively driving penetrative root growth to overcome mechanical impedance (Sadeghi et al., 2014). (2) Concurrent architectural optimization occurred through cortical-to-root diameter ratio modulation and xylem vessel diameter reduction (**Figures 5C, D, F–H**). While this structural adaptation may incrementally elevate radial hydraulic resistance, it concurrently enhances edaphic adaptability by improving axial water transport efficiency and aerenchyma-mediated gas exchange (Ogura et al., 2019).

The experimental results demonstrated that under low initial moisture conditions, compaction treatment enhanced theoretical root hydraulic conductivity in both maize cultivars (**Figure 6**). This finding diverges from the conclusions of Shaheb et al. (2021), who reported reduced root hydraulic conductivity under mechanical compaction in specific contexts. This discrepancy can be attributed primarily to difference in soil moisture status during compaction and the severity of mechanical loading. Northeast China’s black soil, characterized by high organic matter content and low bulk density (Liu et al., 2006), exhibits improved soil structural stability under moderate compaction at suboptimal moisture conditions. Such mechanical compaction reduces interaggregate pore spaces, thereby lowering root-soil interfacial resistance and enhances water acquisition efficiency through optimized capillary connectivity (Liu et al., 2022), which consequently elevates root hydraulic conductivity. Notably, however, our results exhibited congruence with Liu et al.’s studies under high moisture regimes, wherein compaction uniformly suppressed root hydraulic conductivity across cultivars. Under elevated soil moisture, compaction further densifies the soil, reducing porosity and inducing rhizospheric hypoxia. This oxygen deprivation suppressed root respiration and intracellular phosphorylation, impairing aquaporin-mediated water transport, thereby diminishing hydraulic conductivity (Vandeleur et al., 2005). In drought-stressed soils with low bulk density, moderate compaction enhanced water retention and amplified root-soil interfacial contact, facilitating aquaporin-driven osmotic uptake. Conversely, under high moisture (>80% FC), compaction decreased soil permeability, disrupting capillary water mobility and suppressing hydraulic conductivity (Frene et al., 2024, Peralta Ogorek et al., 2025). Given the moisture-dependent duality of compaction effects, site-specific management protocols must dynamically adjust compaction intensity based on real-time soil matric potential thresholds. Overall, these findings demonstrate that the effects of soil compaction on root hydraulic conductivity are highly dependent on soil moisture conditions and compaction intensity, highlighting the need for context specific management.

The root-soil water potential gradient and root xylem bleeding serve as key indicators of root water uptake capacity (Cai et al., 2022). Previous studies have shown that soil compaction can restrict ethylene diffusion and alter phytohormone dynamics, including abscisic acid (ABA) accumulation in the xylem and auxin redistribution within roots, which are associated with changes in root hydraulic plasticity and permeability (Bello-Bello et al., 2022; Pandey et al., 2021). In this study, compaction reduced both the water potential gradient and exudation rate in Zheng 58, yet conversely increased these parameters in Chang 7-2 (**Figure 7**). These contrasting responses suggest genotype-dependent regulatory strategies of root water uptake mechanical stress. Although phytohormone levels were not directly quantified in this study, previous reports of genotype-dependent differences in ABA synthesis in response to soil compaction have been reported previously (Xiong et al., 2020), which may partly account for the divergent root bleeding responses observed between Zheng 58 and Chang 7-2.

Correlation analyses integrating root hydraulic traits, water potential gradients, and anatomical characteristics revealed a strong moisture dependent coordination between root structure and hydraulic function. Uder low initial soil moisture prior compaction, root hydraulic conductivity at both the root-shoot junction and the apical region was positively associated with aerenchyma development and cortex to root diameter ratio, indicating that moderate aerenchyma formation and cortical expansion jointly enhanced hydraulic efficiency by improving internal aeration and structural connectivity (**Supplementary Figure S1A**). In this condition, root water uptake was primarily regulated by anatomical optimization rather than by the root-soil water potential gradient alone (**Supplementary Figure S1B**). In contrast, under initial high soil moisture prior compaction, aerenchyma expansion particularly near the root apex was negatively correlated with hydraulic conductivity, while bleeding sap volume remained strongly driven by the root-shoot soil water potential gradient. The pattern suggest that excessive are aerenchyma formation under compacted and water saturated conditions reflects a stress response to hypoxia rather than a functional enhancement of water transport, as soil densification and reduced pore continuity constrain effective water movement. Collectively, these results demonstrate that the contribution of aerenchyma and cortical structure to root hydraulic performance is highly dependent on soil moisture conditions and root position, highlighting the necessity of integrating anatomical traits with hydraulic drivers when interpreting root water uptake under soil compaction.

Consistent with these hydraulic responses, soil compaction induced pronounces changes in root morphological strategies. When maize roots encounter soil compaction, the proportion of fine roots tends to decreases while root diameter increases, representing a structural adjustment that facilitate penetration into deeper soil lagers and enhance access to water and nutrients under mechanically restrictive conditions. In contrast, a higher proportion fine roots generally increases the root-soil contact interface, thereby improving water and nutrient acquisition efficacy (Correa et al., 2022). However, when compaction occurred under high soil moisture (80% field capacity), the combined effects of elevated mechanical impedance and reduced soil aeration imposed stronger constraints on root elongation and hydraulic function. Therefore, field operations involving heavy machinery should be avoided during high-moisture periods to mitigate adverse effects on root architecture and subsequent crop productivity. From a mechanistic perspective, the observed remodeling of root morphology and hydraulic function may involve the integrational of mechanical sensing, hormonal signaling, and downstream transcriptional regulation. Future studies combining hormonal profiling, mechano-perception analyses, and transcriptomic approaches will be essential to elucidate the upstream regulatory pathways linking soil compaction stress to root anatomical restructuring and hydraulic adjustment, thereby facilitating a transition from phenomenological observations toward a mechanistic understanding.

## 5 Conclusion

1. Under compaction treatment, Chang 7–2 demonstrated superior water absorption capacity relative to Zheng 58, highlighting cultivar-specific physiological adaptations to soil mechanical stress.

2. Differential effects of initial soil moisture content on root morphological responses to compaction: The influence of compaction on maize root systems varied significantly depending on initial soil moisture levels. Under lower initial moisture conditions (40% *FC*), compaction improved root architectural traits by enhancing aerenchyma formation and hydraulic conductivity, thereby promoting root functionality. Conversely, at higher initial moisture (80% *FC*), compaction adversely affected root systems, leading to reduced hydraulic conductivity and impaired water and nutrient uptake efficiency.

3. When field moisture content is high, conducting large-scale mechanized field operations can adversely affect maize root systems. Therefore, agricultural soil management and machinery operation strategies should be adjusted to schedule mechanized operations during periods of lower field moisture content, aiming to mitigate potential impacts on maize root growth.

## Data Availability

The original contributions presented in the study are included in the article/supplementary material. Further inquiries can be directed to the corresponding author/s.

## Supplementary material

Supplementary Figure 1 Correlation network among theoretical root hydraulic traits, root-soil water potential difference, and anatomical characteristics under initial soil water content prior soil compaction. **(A)**, 40% field capacity; **(B)**, 80% field capacity; *RBV*, root bleeding volume; *ΔΨ_RS*, root-shoot water potential difference; *K^t^RSJ*, theoretical root hydraulic conductivity at the root-shoot junction; *K^t^Apex*, theoretical root hydraulic conductivity at 2.5 cm from the root apex; *AA_RSJ* and *AA_Apex*, aerenchyma area at the root-shoot junction and root apex, respectively; *AA_RSJ* and *AA_Apex*, cortical-to-root diameter ratio at the corresponding positions. **p* < 0.05.
