# Supplementary material for: Impact of soil compaction degree modulated by initial water content on maize root phenotype and hydraulic properties
Source: Front Plant Sci. 2026 Feb 11;17:1764502. doi: 10.3389/fpls.2026.1764502 (PMC12932624; doi:10.3389/fpls.2026.1764502)
Supplement: Supplementary Figure 1 — Correlation network among theoretical root hydraulic traits, root-soil water potential difference, and anatomical characteristics under initial soil water content prior soil compaction. (A), 40% field capacity; (B), 80% field capacity; RBV, root bleeding volume; ΔΨ_RS, root-shoot water potential difference; KtRSJ, theoretical root hydraulic conductivity at the root-shoot junction; KtApex, theoretical root hydraulic conductivity at 2.5 cm from the root apex; AA_RSJ and AA_Apex, aerenchyma area at the root-shoot junction and root apex, respectively; AA_RSJ and AA_Apex, cortical-to-root diameter ratio at the corresponding positions. *p < 0.05. [file Supplementaryfile1.docx]

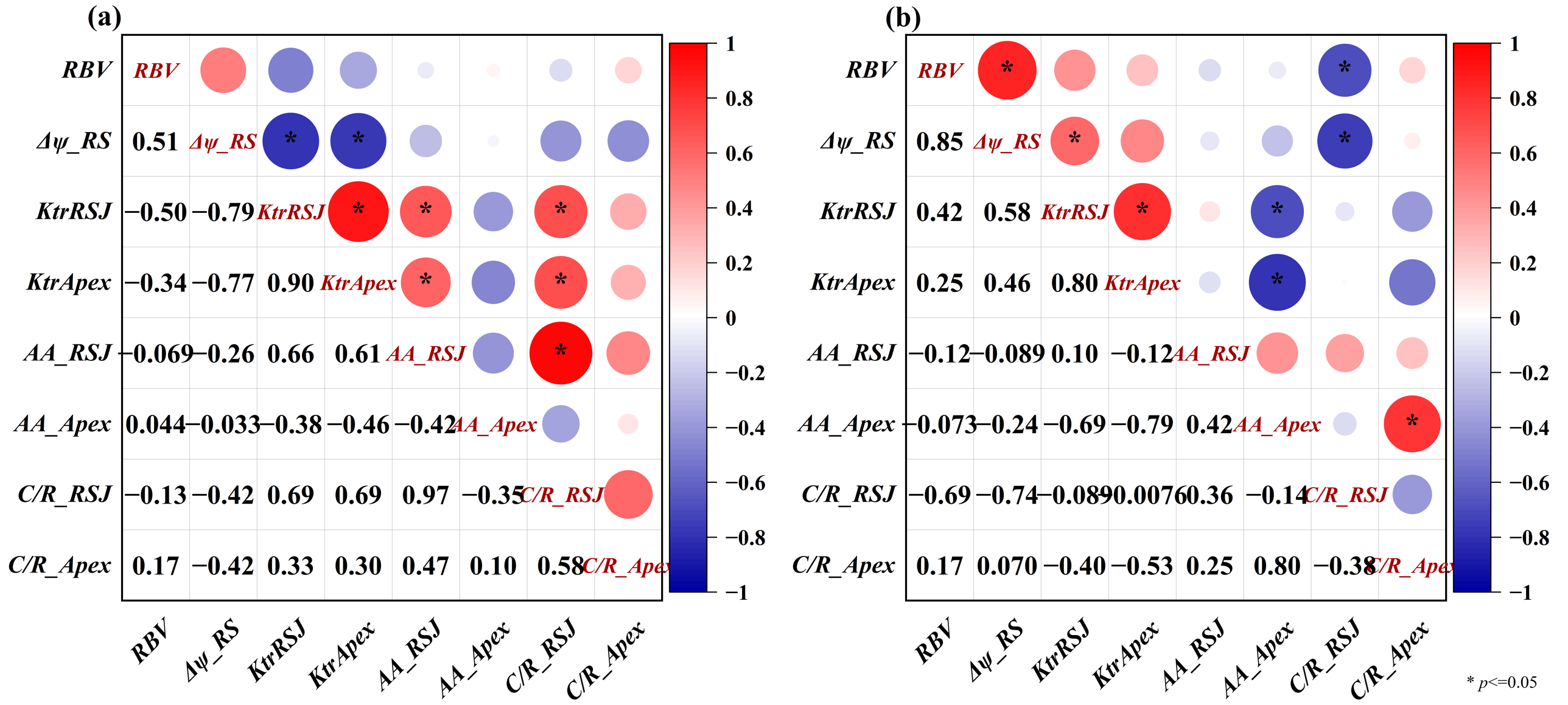


Fig. S1 Correlation network among theoretical root hydraulic traits, root-soil water potential difference, and anatomical characteristics under initial soil water content prior soil compaction. (a),40% field capacity; (b),80% field capacity; *RBV*, root bleeding volume; *ΔΨ_RS*, root-shoot water potential difference; *KₜRSJ*, theoretical root hydraulic conductivity at the root-shoot junction; *KₜApex*, theoretical root hydraulic conductivity at 2.5 cm from the root apex; *AA_RSJ* and *AA_Apex*, aerenchyma area at the root-shoot junction and root apex, respectively; *AA_RSJ* and *AA_Apex*, cortical-to-root diameter ratio at the corresponding positions. **p* < 0.05
